# Type 2 Diabetes Mellitus and Macrovascular Complications

**DOI:** 10.1155/2017/4301461

**Published:** 2017-10-31

**Authors:** Peng-Fei Shan, Qian Li, Mogher Khamaisi, Gui-fen Qiang

**Affiliations:** ^1^Department of Endocrinology and Metabolism, The Second Affiliated Hospital, Zhejiang University School of Medicine, Hangzhou, Zhejiang, China; ^2^Research Division, Joslin Diabetes Center, Harvard Medical School, Boston, MA, USA; ^3^Division of Internal Medicine, Rambam Health Care Campus and Faculty of Medicine, Technion-Israel Institute of Technology, Haifa, Israel; ^4^Institute of Endocrinology, Diabetes & Metabolism, Rambam Health Care Campus, Haifa, Israel; ^5^Institute of Materia Medica, Chinese Academy of Medical Sciences & Peking Union Medical College, Beijing, China; ^6^Department of Physiology & Biophysics, College of Medicine, University of Illinois at Chicago, Chicago, IL, USA

The number of people with type 2 diabetes mellitus (T2DM) has been increasing worldwide due to aging, urbanization, dietary, and lifestyle changes. It was estimated that there were 415 million people with diabetes aged 20–79 years in 2015, and the number was predicted to rise to 642 million by 2040 [1]. With comprehensive control of risk factors of cardiovascular disease (CVD), there has been a decline in prevalence and mortality from diabetic macrovascular complications over the past several decades in some western countries including United States of America. Macrovascular complications, mainly including cardiovascular and cerebrovascular diseases, are still the most common complications and the major cause of mortality and morbidity in patients with T2DM.

In recent years, there are several advances in this field. Diabetes patients usually have hyperglycemia, hyperlipidemia, and insulin resistance; all of them are risk factors for macrovascular diseases. PKCs, RAGE, and ROS may mediate the effects of hyperglycemia and hyperlipidemia on cardiovascular systems. Knockout of PKC*β*, RAGE, and Nox1 could attenuate the development of atherosclerosis in diabetic mice [2, 3]. To mimic endothelial or macrophage insulin resistance, insulin receptor was specially knocked out in endothelial cells or macrophages. The development of atherosclerosis was accelerated in endothelial specific insulin receptor knockout mice, and the plaque was more unstable in macrophages-specific insulin receptor knockout mice. Look AHEAD trial and the Italian Diabetes and Exercise Study indicate that lifestyle management significantly improves physical fitness, A1c, and coronary heart disease (CHD) risk factors. Weight loss surgery, especially bariatric surgery, could result in weight loss, A1c improvement [4], and CVD outcome improvement. Furthermore, data are available about the effect of glucose-lowering therapies on cardiovascular risk in patients with T2DM. In addition, clinic trials have demonstrated that with comprehensive management of CVD risk, factors such as lowering blood glucose, smoking rate, and total cholesterol as well as blood pressure mortality rates from macrovascular complications have been declining steadily in some countries.

In this issue, T. Hu et al. found that decreased expression of CSE and 3-MST and the subsequently decreased production of hydrogen sulfide (H_2_S) contributed to high glucose- (HG-) induced sFlt-1 production via activating ADAM17 in adipocytes. Exogenous H_2_S donor NaHS has a potential therapeutic value for HG-induced sFlt-1 production. S. Ye et al. found that the traditional Chinese medicine Didang Decoction intervention could upregulate the expression of cAMP-1 and PKA and downregulate the expression level of MLCK and PKC, thus ameliorating vascular endothelium injury in high-fat diet followed by the small dose of STZ-induced diabetic animal model.

T2DM is always accompanied by a cluster of risk factors of CVD such as high blood pressure, dyslipidemia, central obesity, and albuminuria [5]. M.-F. Yao et al. explored the differences in the risks of CHD and stroke between Chinese women and men with T2DM and their association with metabolic syndrome in Hangzhou, China. They found that women had lower CHD risk, fatal CHD risk, stroke risk, and fatal stroke risk compared with men with T2DM. The CHD risk was significantly higher in men with MS than in those without MS; and the CHD and stroke risks were higher in women with MS than in those without MS. In a total of 9473 subjects aged over 45 years, including 1648 patients with T2DM, X. Ding et al. found that the patients with prediabetes or T2DM were with higher risks to have hypertension. T2DM with nonalcoholic fatty liver disease (NAFLD) had significantly higher levels of blood pressure, triglyceride, uric acid, and HOMA-IR than those without NAFLD. Hypertriglyceridemia, NAFLD, hyperuricemia, and insulin resistance were associated with a higher prevalence of hypertension independent of other metabolic risk factors in type 2 diabetes. B. Stratmann et al. found that serum phospholipid and serine levels independently discriminated T2D patients with and without CAD. Oxidative stress, which is increased in T2D, leads to profound changes in the content and composition of biological membranes and accelerated phospholipid degradation, resulting in lower metabolite levels of PCs and serine.

Lower extremity peripheral arterial disease (PAD) is a common type of PAD in patients with T2DM, which can be noninvasively and objectively diagnosed by using the ankle-brachial index (ABI), and is also a marker of arterial atherosclerosis at other sites [6]. J. Ma et al. evaluated the concordance between oscillometric ABI and standard Doppler ABI in diabetic patients with or without diabetic foot. They found that the oscillometric measurement was proven to be a reliable, convenient, and less time-consuming alternative to standard Doppler ABI in patients. X.-H. Pang et al. determined the relationship between lower extremity peripheral arterial disease (PAD), 10-year CHD, and stroke risks in patients with type 2 diabetes. They found that 88 (7.5%) patients were detected with PAD in 1178 T2DM patients. ABI was an independent predictor of 10-year CHD and stroke risks in T2DM patients. Compared with those in the T2DM non-PAD group, the odds ratios (ORs) for CHD and stroke risk were 3.6 (95% confidence interval (CI), 2.2–6.0; *P* < 0 001) and 6.9 (95% CI, 4.0–11.8; *P* < 0 001) in those with lower extremity PAD, respectively.

Coronary revascularization is a safe and effective therapy for patients with single and multivessel coronary artery disease. Patients with diabetes mellitus contribute to about 25% of patients undergoing coronary revascularization [7]. Coronary artery bypass grafting (CABG) surgery is considered a standard of care for patients with advanced coronary artery disease. The optimal glycosylated hemoglobin (HbA1C) target may be especially pertinent in diabetic patients with coronary artery diseases. J. Zheng et al. made a meta-analysis and systemic review including 7895 diabetic patients undergoing coronary artery bypass grafting surgery from eight published studies. Combined analyses revealed that higher HbA1c level was significantly associated with increased risks of all-cause mortality, myocardial infarction, and stroke in diabetic subjects undergoing CABG surgery.

In conclusion, there is ongoing progress in the pathophysiology, glycemia control, and risk factors of diabetic macrovascular complications shown in this special issue. Several other fields are also showing progress, such as weight loss surgery, but these are not discussed in the present issue. We believe that the present issue could bring some latest progress in this field and will be of interest to the readers.
